# Arne Schousboe: In Memoriam (1944–2024)

**DOI:** 10.1007/s11064-024-04136-6

**Published:** 2024-03-28

**Authors:** Henry Sershen

**Affiliations:** Nathan S. Kline Institute for Psychiatric Research, Orangeburg, USA

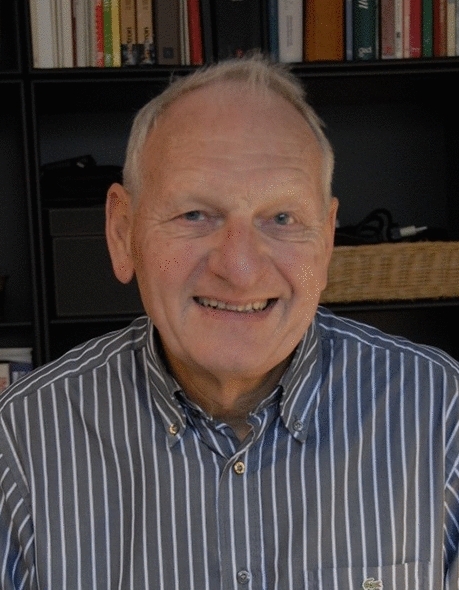



The journal (Neurochemical Research), and publisher Springer, would like to express its sadness and condolences in the passing of a dear friend and our Editor-in-Chief (EIC) Professor Arne Schousboe. Arne was a long-time member of the journal’s editorial board and became its Editor-in-Chief 12 years ago (2011). He also contributed to the journal very early on and published one of its first articles with Leif Hertz and Gerd Svenneby on the uptake and metabolism of GABA in the second volume of the journal (Neurochem. Res., Vol. 2(2) 217–29, 1977). A special issue of the journal was assembled in his honor as a tribute to his scientific contributions (Neurochem. Res., Vol. 27, Nos. 1/2, February 2002). As EIC, he added the current structure for handling the increasing number of submissions from around the world, adding a dedicated group of Associate Editors, and always striving to attract high quality manuscripts and to improving the impact factor of our journal.

I’m sure his numerous publications and contributions to the scientific community will be conveyed by the many neurochemical societies that he was a member and even organizer (ASN, ESN, ISN, NTS, and the ICBEM meetings) during the past + 50 years, as they express this sad loss. In this note, we would like to thank him more for his personal side—as a friend and colleague. I would like to thank him for his long friendship, as a colleague in research during the times we discussed science and importantly his strong support of our journal.

Our Associate Board members also reflected some similar personal thoughts—“He will aways be remembered not only by his many achievements, but also by the nicest person he was.”—“His kindness.”—“Arne was a great neurochemist and an outstanding human being. I will keep forever the memories of the time I spent in his lab in Copenhagen in the late 90's and his great sense of humor.”—“He was a gentleman’s gentleman. Always greets you with a smile, a modest person, and an excellent scientist.”—“Arne will be kindly remembered by all of us who had the privilege to know him and work with him. This is indeed very sad loss.”—“He was a classic gentleman. A fountain of knowledge, yet always humble, modest, respectful, intuitive, careful listener, and on and on. Such a wonderful ambassador for science and humanity. He will be missed indeed.”

- the neuroscience community loses one of its leading members.

*Henry Sershen*

Managing Editor.

*Carlos Duarte, Joao Duarte, Gustavo da Costa Ferreira, Laszlo G. Harsing, Jr., Cheng He, Hiroshi Katsuki* *,* *Jarogniew Łuszczki, Arturo Ortega, Albert C.H. Yu, Maarten Reith and Michael Aschner and Naren Banik.*

Associate Editors.

*Greg Baer*

Springer Nature.

